# Improved Astaxanthin Production with *Corynebacterium glutamicum* by Application of a Membrane Fusion Protein

**DOI:** 10.3390/md17110621

**Published:** 2019-10-31

**Authors:** Nadja A. Henke, Volker F. Wendisch

**Affiliations:** Genetics of Prokaryotes, Faculty of Biology & CeBiTec, Bielefeld University, P.O. Box 10 01 31, 33501 Bielefeld, Germany; n.henke@uni-bielefeld.de

**Keywords:** astaxanthin, *Corynebacterium glutamicum*, fusion protein, β-carotene ketolase, β-carotene hydroxylase

## Abstract

Astaxanthin is one of the strongest natural antioxidants and a red pigment occurring in nature. This C40 carotenoid is used in a broad range of applications such as a colorant in the feed industry, an antioxidant in cosmetics or as a supplement in human nutrition. Natural astaxanthin is on the rise and, hence, alternative production systems are needed. The natural carotenoid producer *Corynebacterium glutamicum* is a potent host for industrial fermentations, such as million-ton scale amino acid production. In *C. glutamicum*, astaxanthin production was established through heterologous overproduction of the cytosolic lycopene cyclase CrtY and the membrane-bound β-carotene hydroxylase and ketolase, CrtZ and CrtW, in previous studies. In this work, further metabolic engineering strategies revealed that the potential of this GRAS organism for astaxanthin production is not fully exploited yet. It was shown that the construction of a fusion protein comprising the membrane-bound β-carotene hydroxylase and ketolase (CrtZ~W) significantly increased astaxanthin production under high glucose concentration. An evaluation of used carbon sources indicated that a combination of glucose and acetate facilitated astaxanthin production. Moreover, additional overproduction of cytosolic carotenogenic enzymes increased the production of this high value compound. Taken together, a seven-fold improvement of astaxanthin production was achieved with 3.1 mg/g CDW of astaxanthin.

## 1. Introduction

**Figure 1:**
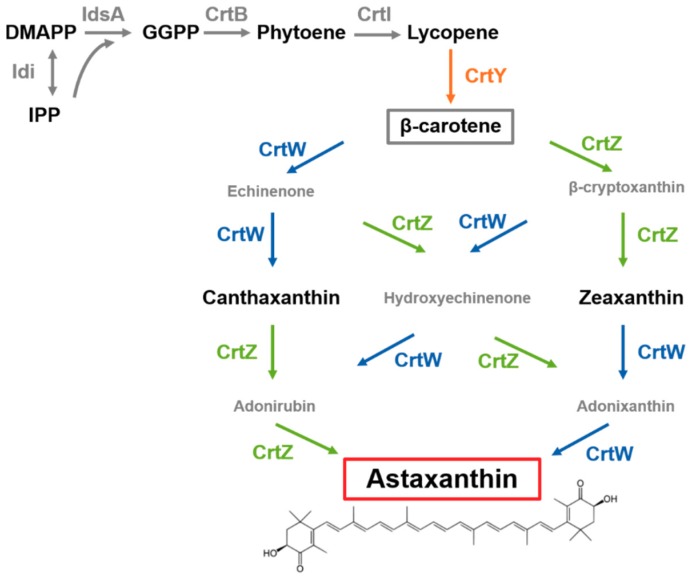
Astaxanthin biosynthesis in engineered *C. glutamicum*. Astaxanthin biosynthesis with DMAPP (dimethylallyl pyrophosphate) and IPP (isopentenyl pyrophosphate) as precursors involves seven enzymes: endogenous Idi: isopentenyl pyrophosphate isomerase, IdsA: GGPP synthase, CrtB: phytoene synthase and CrtI: phytoene desaturase; as well as heterologous CrtY: lycopene cyclase, CrtW: β-carotene ketolase and CrtZ: β-carotene hydroxylase.

The demand for naturally produced astaxanthin is estimated to witness an exponential growth of 25% (CAGR) by 2025 [1]. This fact is in accordance with a worldwide trend: consumers seek for natural products that are produced in an environmentally friendly way. Moreover, the feed industry, which is the main purchaser of astaxanthin, is growing as well in order to satisfy the demand for animal-based food products such as meat, fish and eggs [2]. As the astaxanthin market is dominated by synthetic astaxanthin that is made from petro chemistry, new production capacities are needed to meet the increasing demand for naturally produced astaxanthin [1]. Fermentations with *Corynebacterium glutamicum* dominate food and feed biotechnology since decades for the production of amino acids e.g. l-glutamate and l-lysine [3,4,5]. This microorganism naturally synthesizes carotenoids as pigments and yet investigations concerning this output are limited. The carotenoid/terpenoid precursors dimethylallyl pyrophosphate (DMAPP) and isopentenyl pyrophosphate (IPP) are derived from the MEP-pathway in this organism [6]. Carotenoid biosynthesis of the natural C50 carotenoid decaprenoxanthin is based on the accumulation of the C40 intermediate lycopene [7,8]. The genome of *C. glutamicum* contains a major carotenoid operon [9] and the regulatory mechanism involving a MarR-type transcriptional regulator CrtR was identified [10]. Interestingly, this regulation is working in a metabolite dependent manner in which the carotenoid precursor molecule geranylgeranyl pyrophosphate (GGPP) is acting as an effector of the repressor CrtR [10]. Thus, as the genetic background was analyzed in more detail, the production of industrially relevant carotenoids of the C40 family was conducted over the last years [6,7,11] (Figure 1). Indeed, metabolic engineering studies showed that *C. glutamicum* is a suitable host for production of various C40 and C50 carotenoids [11,12] with astaxanthin as prominant example [13]. The previously published strain ASTA1 possesses a good volumetric productivity, but for industrial scale production further improvement is needed in order to meet economically feasible production titers. 

The construction of fusion proteins and the application of membrane anchors was shown to be an efficient strategy to increase membrane integrity and protein stability. It was shown that linkage of CrtW and CrtZ with the GlpF protein from *E. coli* increased astaxanthin production by approximately 2-fold [14]. Moreover, the fusion of a geranylgeranyl pyrophosphate synthase with a phytoene synthase improved carotenoid production in plant cells [15]. Recently, it was shown that fusion of the terminal carotenoid enzymes increased astaxanthin production in *E. coli* [16].

**Figure 2:**
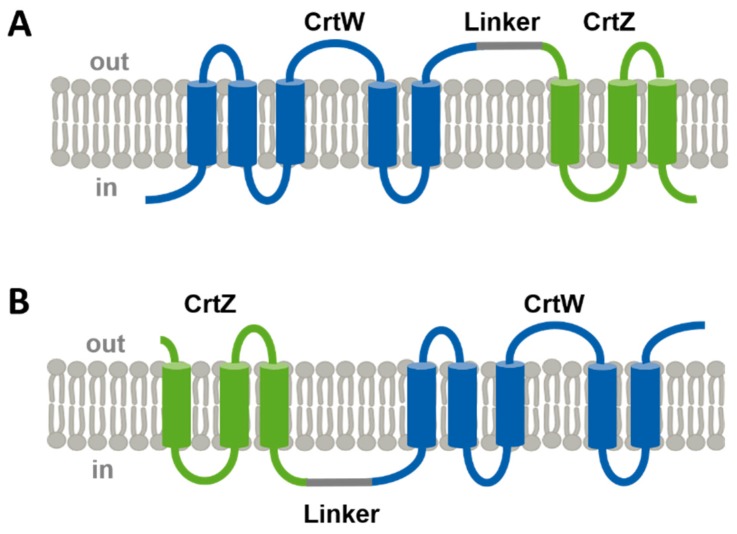
Scheme of newly constructed fusion proteins CrtW~Z (**A**) and CrtZ~W (**B**). Transmembrane helices were predicted with TMHMM [17]. CrtW comprises 5 TMH with an intracellular N-terminus; CrtZ comprises 3 TMH with an extracellular N-terminus; both fusions contained an identical synthetic linker sequence of 10 amino acids (GGGGSGGPGS).

In this study, a fusion protein strategy was conducted (Figure 2) that resulted in an approximately five-fold improved astaxanthin production under high glucose concentration in *C. glutamicum*. Moreover, it turned out that the co-utilization of glucose and potassium acetate significantly improved astaxanthin production in small-scale fermentations. Thus, not only classical metabolic engineering strategies, but also the construction of fusion proteins and the optimization of cultivation media are powerful strategies to exploit the potential of valuable compound formation. 

## 2. Results

### 2.1. Fusion of the Membrane-bound Proteins β-carotene Hydroxylase and β-carotene Hydroxylase Increased Astaxanthin Production under High Glucose Concentration

**Figure 3:**
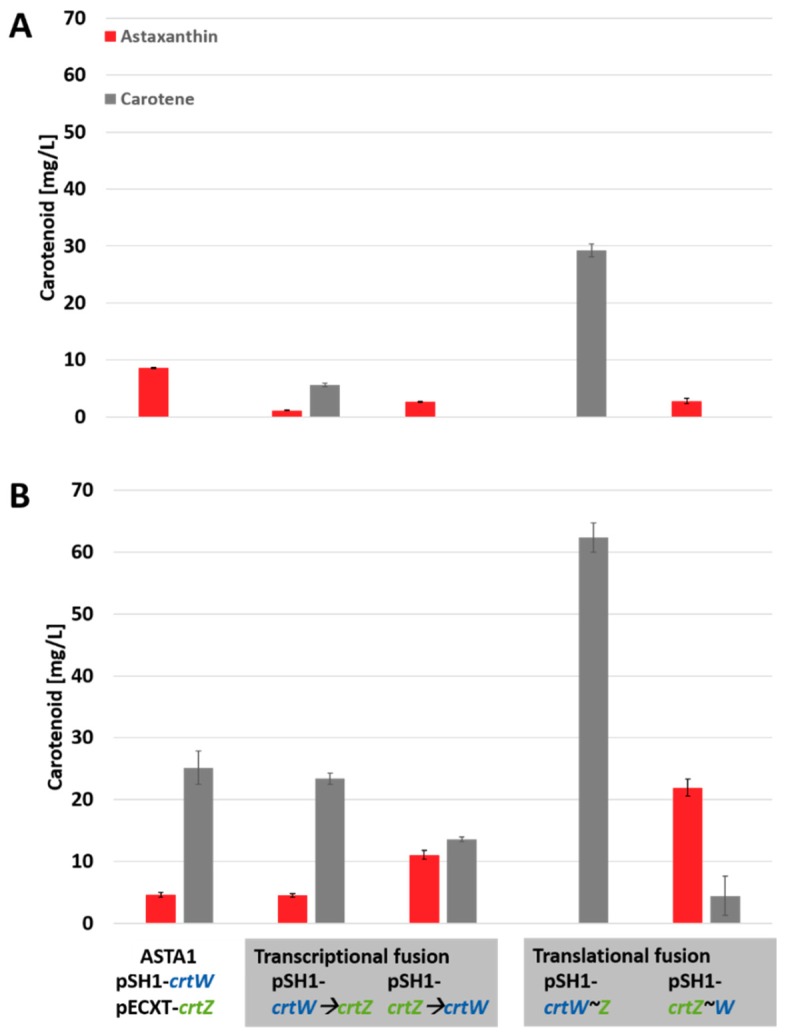
Production profiles of engineered *C. glutamicum* strains for astaxanthin production under (**A**) low (2%) and (**B**) high (4%) glucose concentration. Product titers (in mg/L) were determined from three biological triplicates after cultivation for 48 h in biolector microcultivation system.

Previous studies have shown that astaxanthin biosynthesis in engineered *C. glutamicum* strains is limited by the conversion of β-carotene to astaxanthin and/or the overall flux towards carotenoid products [13]. The previously constructed ASTA1 strain that carries the expression vectors pSH1-crtW and pECXT-crtZ yielded astaxanthin as the major carotenoid product under low glucose concentration [13] as shown in Figure 3. However, it turned out that the astaxanthin production in this strain is not robust to higher glucose concentration (Figure 3). Since β-carotene accumulated, it is tempting to speculate that the two functionalizing enzymes CrtW and CrtZ are limiting under such cultivation conditions, which are favorable for industrial applications in order to achieve high cell densities. 

Both enzymes, β-carotene hydroxylase and β-carotene ketolase, are predicted to be transmembrane enzymes with three (CrtZ) and five (CrtW) putative transmembrane helices [17]. As heterologous overproduction of transmembrane enzymes is critical, here a transcriptional and a translational fusion strategy approach were applied in order to facilitate protein stability, membrane integrity and intermediate channeling. The construction of artificial operon crtW→Z and crtZ→W yielded astaxanthin production whereas the crtZ→W construct already doubled astaxanthin production under high glucose concentration (Figure 3). Moreover, the two enzymes CrtW and CrtZ were translationally fused with an artificial ten amino acid linker sequence (GGGGSGGPGS) and in both sequential orders (Figure 2). It turned out that the protein fusion CrtW~Z did not result in astaxanthin production presumably due to an inactive fusion enzyme. In contrast, a functional CrtZ~W fusion protein resulted in an about five-fold increased astaxanthin accumulation in comparison to the two-vector system in ASTA1 (Figure 2 and Figure 3). 

All constructed one-vector systems, including the fusion proteins were cultivated under low (2%) and high (4%) glucose concentration and compared to the previously published two-vector system. Under low glucose concentration, the two-vector system pSH1-crtW & pECXT-crtZ yielded the highest astaxanthin titer of 8.6 mg/L (1.3 mg/g CDW). Concerning the one-vector system, all constructs yielded astaxanthin accumulation except for the fusion protein construct pSH1-crtW~Z (Figure 3). However, under this condition all newly constructed strains resulted in a decreased astaxanthin titer when compared to the two-vector system. However, under high glucose concentration, which is favorable for industrial applications to yield high biomass titers, the two-vector system yielded only 50% of the astaxanthin compared to the low glucose concentration (Figure 3). However, the one vector system comprising the transcriptional fusion of crtZ and crtW resulted in increased astaxanthin titers as the transcriptional fusion strain BETA4 (pSH1-crtZ-crtW) produced 11 mg/L (0.42 mg/g CDW) astaxanthin. Moreover, the translation fusion strain BETA4 (pSH1-crtZ~W) was the best strain as it produced 22 mg/L (1.7 mg/g CDW) of astaxanthin, which was almost five-fold better than the two-vector system (Figure 3). This strain was named ASTA*. Thus, it turned out that the fusion protein strategy considerably affected the astaxanthin titer under high glucose concentration in fermentations of *C. glutamicum* strains.

### 2.2. Overproduction of Cytosolic Carotenoid Enzymes Increased Astaxanthin Production under High Glucose Concentration

**Figure 4:**
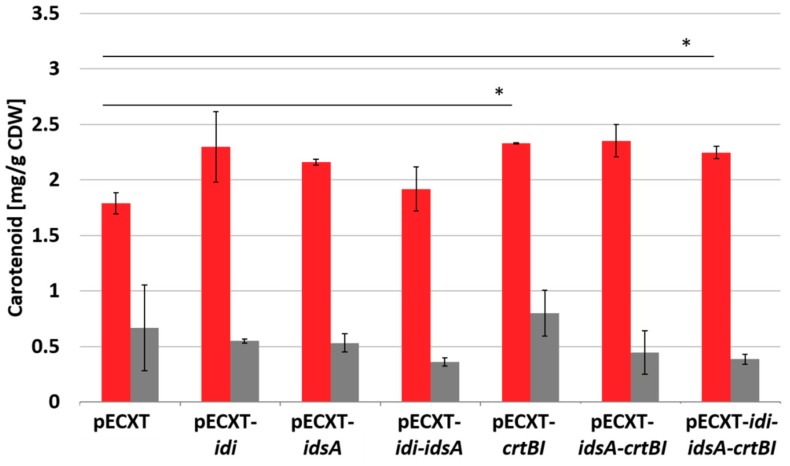
Effect of overexpression of genes for cytosolic carotenoid biosynthesis enzymes in *C. glutamicum* ASTA*. Astaxanthin (red) and β-carotene (grey) content is given in mg/g CDW from 48 h cultivations in CGXII medium with 4% glucose from biolector microcultivation system. Mean values and standard deviations are given. Significance was calculated with a students’ t-test, *p* < 0.05.

As the strain ASTA* produced astaxanthin as the dominant carotenoid under high glucose concentration (Figure 3), but did not reach maximal β-carotene titers, other upstream working enzymes of the carotenoid pathway may have limited astaxanthin production under these conditions. Therefore, it was tested whether other carotenogenic enzyme(s) limit the flux towards astaxanthin in the newly constructed ASTA* strain. Hence, the genes *idi*, *idsA* and *crtBI* coding for isopentenyl pyrophosphate isomerase, GGPP synthase, phytoene synthase and phytoene desaturase were overexpressed in ASTA*. As shown in Figure 4, the astaxanthin content slightly increased in all constructed strains. However, only combined overexpression of all tested genes (*idi*, *idsA* and *crtBI*) or of phytoene synthase and desaturase genes (*crtBI*) significantly increased the astaxanthin titer to 2.25 ± 0.06 mg/g CDW, which is about 25% more than the production of the control strain (1.8 ± 0.1 mg/g CDW) (Figure 4). Thus, it could be shown that overexpression of genes encoding for upstream and cytosolic carotenoid biosynthesis affected astaxanthin production positively and represent new targets for chromosomal engineering of the platform strain. 

### 2.3. Co-utilization of Glucose and Potassium Acetate Improved Astaxanthin Production in Minimal Medium

**Figure 5:**
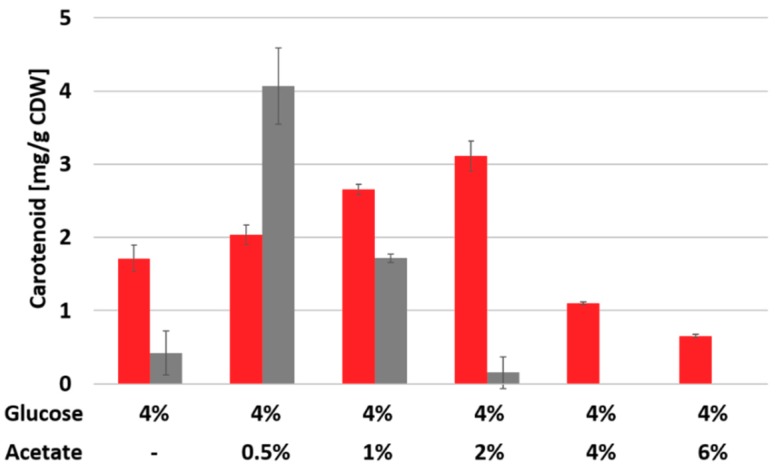
Astaxanthin production by strain ASTA* from blends of glucose and acetate. Astaxanthin (red) and β-carotene (grey) production are given in mg/g CDW from a 48 h cultivation in CGXII medium with 4% glucose plus different amounts of potassium acetate from cultivation in biolector microcultivation system. Mean values and standard deviations are given.

Based on our findings on engineering carotenogenesis downstream of the central precursors IPP and DMAPP, we postulated that the overall bottleneck for astaxanthin production in the ASTA* strain might be the central carbon fluxes and not only the expression of carotenogenic genes. Since the MEP pathway draws pyruvate and GAP from glycolysis and since it is known that during growth on mixtures of glucose and potassium acetate the *C. glutamicum* cell accumulates glycolytic intermediates to higher concentrations [18], a cultivation using a blend of the two carbon sources glucose and potassium acetate was performed (Figure 5). Indeed, it could be shown that addition of up to 2% potassium acetate in the CGXII medium together with 4% glucose significantly increased the overall production titer. Under conditions were 4% glucose and 2% acetate were utilized the maximal titer of 3.11 ± 0.2 mg/g CDW astaxanthin were reached which is 80% more than the production from glucose as sole carbon source (1.72 ± 0.18 mg/g CDW) (Figure 5). However, when the acetate concentration was further increased to 4% and 6%, astaxanthin production significantly dropped to about 1 and < 1 mg/g CDW, respectively (Figure 5).

## 3. Discussion

In this work, astaxanthin production in *C. glutamicum* was improved through the application of three different strategies, namely: construction of membrane fusion-proteins, overproduction of cytosolic carotenoid biosynthesis enzymes and use of blends of glucose and potassium acetate as fermentation substrates. Astaxanthin production with *C. glutamicum* is of high interest as this workhorse is used in a safe manner for more than 60 years in the food and feed industry. Although *C. glutamicum* can grow to high cell densities of up to 95 g/L [19,20], fermentation processes with *C. glutamicum* for secreted product such as amino acids are characterized by a high proportion of substrate being converted to product rather than to biomass [19]. It has been shown that *C. glutamicum* is a potent host for high titer terpenoid production as maximal titers of 1.25 g/L of secreted isopentenol were reached [21]. We have previously shown that multiple products can be coproduced, i.e., cell-bound carotenoids with secreted amino acids [22]. However, lysine is not produced by wild type based strains as the parental strain of this study (only the astaxanthin and lysine producing recombinant ASTALYS produced lysine). Thus, we did not find lysine in supernatants of the astaxanthin producing strains used here (data not shown). By contrast, glutamate production can be triggered in the wild type. Glutamate could be coproduced with astaxanthin by recombinants based on wildtype [22]. Interestingly, some glutamate was produced by the astaxanthin strain ASTA1 even in the absence of any trigger [22], which we could confirm here. The application of the fusion protein CrtZ~W resulted in maximal glutamate titers of around 7 mM (data not shown), which are in accordance with previously published data obtained with the ASTA1 strain [22].

The production process for astaxanthin, a cell-bound product, requires a high cell density cultivation in order to reach economically feasible productivites. For this reason, the robustness of the astaxanthin production under high glucose concentration was addressed with the first strategy: the construction of the fusion protein CrtZ~W. 

It was shown that the ASTA* strain with the fusion protein CrtZ~W performed robust in terms of astaxanthin production titers of 4% glucose whereas the forerunner strain ASTA1 comprising single enzymes CrtZ and CrtW did not perform well under such conditions. The explanation for this and related findings on synthetic fusion proteins has not yet been analyzed well. However, it is typically argued that fusion proteins, in a similar manner as application of protein scaffolds and colocalization in compartments, result in an optimized transfer of intermediates between enzymes [23,24,25]. Application of fusion proteins for carotenoid production has recently gained more interest. It has been shown that application of a tridomain protein comprising CrtB, CrtI and CrtY doubled β-carotene production by *S. cerevisiae* [26]. Both, the domain order and the linker properties, were claimed to influence the stability and/or expression of such enzymes [26]. Interestingly, the natural astaxanthin producer *Xanthophyllomyces dendrorhous* possesses bifunctional enzymes in carotenogenesis. A fused phytoene synthase-lycopene cyclase CrtYB is encoded in its genome as well as an astaxanthin synthase CrtS that catalyses both ketolation and hydroxylation of β-carotene [27,28]. Moreover, application of such natural or synthetic bifunctional enzymes has not only been proven to accelerate carotenoid production in unicellular systems, but also in plants [29]. In addition, the fusion of a FPP synthase with a patchoulol synthase also accelerated formation of the sesquiterpene patchoulol in yeast [30]. The CrtZ~W fusion protein described here (Figure 2) was constructed with a Gly-Ser rich linker sequence. Such linkers are generally regarded as flexible whereas amino acids Glu, Ala, Lys tend to build rigid linkers [31]. It has to be examined how a rigid linker might influence the CrtZ~W fusion. Besides the flexibility also the length of the linker sequence affects the activity of the enzymes. The ten amino acid linker used in this study is regarded as medium sized, whereas 5 and 21 amino acid linker sequences may be categorized as small and large, respectively [31,32]. Since it was shown that the optimal linker length relies on the fused proteins themselves [16], it has to be validated in the future if shorter or longer linkers might increase astaxanthin production catalyzed by the bifunctional CrtZ~W fusion further. 

In contrast, changing the order of CrtW and CrtZ in the fusion protein did not lead to astaxanthin production, which is most probably due to reduced β-carotene hydroxylase activity of the CrtW~Z fusion protein since neither zeaxanthin nor astaxanthin were synthesized (Figure 3). However, the strain with the CrtW~Z fusion protein produced about 62 mg/L of the precursor β-carotene, which is less compared to about 85 mg/L produced by the parental strain BETA4 (data not shown). Thus, we hypothesize that functional expression of crtW and/or crtZ limits carotenoid biosynthesis. As in other recombinant microorganisms the molecular cause remains to be identified. Moreover, the ketolase activity of fusion protein CrtW~Z might also be limiting in this fusion construct as only minor amounts of canthaxanthin were identified (data not shown). Similarly, a recent study showed that fusion proteins with CrtZ at the N-terminus are superior over N-terminal CrtW fusion proteins as it was demonstrated for different bifunctional enzymes in *E. coli* [29] and *Nicotiana benthamiana* [16]. As an explanation the locatization of histidine motifs is discussed, as these are on the surface of active CrtZ~W fusions, but are inside the inactive CrtW~Z fusion proteins [16]. For an algal β-carotene ketolase it was shown that both N-terminal fusion of the signal peptide from OmpF and C-terminal fusion of TrxA increased astaxanthin formation most likely by a better guiding of the protein to the membrane (OmpF signal peptide) and increased protein stability (TrxA) [33].

The overexpression of cytosolic carotenoid biosynthesis genes only increased astaxanthin production to a minor extent. However, the screening of potential bottleneck enzymes Idi, IdsA, CrtB and CrtI from the upstream carotenoid biosynthesis pathway lead to the conclusion that astaxanthin biosynthesis in ASTA* can be increased by overexpression of crtBI in particular. It is known from a wide number of publications that the carbon source significantly influences the overall metabolic flux and therefore affects product formation by *C. glutamicum* [18]. Glucose and acetate are typically co-utilized in *C. glutamicum* as it was shown in previous studies [34]. Here, co-utilization of 4% glucose with 2% acetate significantly increased astaxanthin titers to more than 3 mg/g CDW which is the highest titer for this organism so far. This effect might be explained by altered concentrations of central metabolites such as GAP and pyruvate, which are the substrates of the MEP-pathway in *C. glutamicum*. Alternatively, as NAD(P)H and 2-oxoglutarate are believed to be cofactors or co-substrates for the ketolation and hydroxylation reactions catalyzed by CrtW and CrtZ, their availability might be affected by the used carbon source [35,36]. It was shown in *E. coli* that co-utilization of glycerol and glucose in a 4:1 ratio is favorable for astaxanthin production over utilization glycerol alone [16]. It was hypothesized that glycerol with a higher average degree of reduction per carbon can provide more reducing equivalents for astaxanthin biosynthesis [16,33,37]. As it turned out that solely the carbon source composition strongly affected astaxanthin production in *C. glutamicum*, further media optimization has to be performed. The basic medium used in this study is the CGXII minimal medium which was initially optimized for amino acid production with an elemental composition of C:N:P of 40:10:1 [38]. Thus, for the production of astaxanthin a DOE approach would be suitable to optimize the minimal medium fundamentally. Moreover, it can be concluded that the potential of *C. glutamicum* has a host for membrane-bound carotenoids is not fully tapped yet and that media composition as part of the process design can make a significant contribution towards economically feasible product titers.

## 4. Materials and Methods 

### 4.1. Bacterial Strains and Growth Conditions

Strains and plasmids used in this study are listed in Table 1. All production experiments were carried out in the BETA4 platform strain [13] that was constructed on the basis of a prophage-cured *C. glutamicum* MB001 [39]. Precultures of *C. glutamicum* strains were grown in complex medium Luria Broth (LB) or Brain Heart Infusion (BHI) supplemented with 2% glucose over night. Main cultures were grown in CGXII minimal medium supplemented with glucose and/or potassium acetate after washing in the minimal medium. Each culture was inoculated to an initial OD_600_ of 1. Cultivations were performed in 1 mL in the Biolector® flowerplate microcultivation system (m2p-labs GmbH, Baesweiler, Germany) at 1100 rpm at 30 °C. *E. coli* DH5α cells were cultivated at 37 °C in LB medium. Tetracycline and kanamycine were added if appropriate in concentrations of 5 and 25 µg mL^−1^. 

### 4.2. Cloning of Expression Vectors

Expression plasmids were constructed in *E. coli* DH5α. First target genes were amplified by a high-fidelity PCR (All-in HiFi, highQu, Kraichtal, Germany) and cloned into digested expression vectors by Gibson-Assembly [45]. Used oligonucleotides are listed in Table 2 and were delivered by Metabion (Planegg/Steinkirchen, Germany). PCR products were purified with PCR- and gel extraction kit (Macherey-Nagel, Düren, Germany). *E. coli* DH5α cells were transformed by heat shock after preparation of CaCl_2_ competent cells. Transformants were screened by colony-PCR and plasmids were isolated by plasmid miniprep kit (GeneJET, Thermo Fisher Scientific, Schwerte, Germany). New expression vectors were confirmed by sequencing. *C. glutamicum* cells were transformed by electroporation as described elsewhere [46]. 

### 4.3. Construction of pSH1- and pECXT99A-based Expression Vectors

For construction of expression plasmids pSH1 and pECXT99A vectors were digested with BamHI (Thermo Scientific Fisher, Schwerte, Germany) and dephosphorylated (Antarctic phosphatase, New England Biolabs, Frankfurt, Germany). PCR products were amplified using the oligonucleotides as the following: *crtW1*: FpW1 + HN05; *crtZ1*: HN06 + HA35; *crtW-L*: FpW1 + HA47; *L-crtZ*: HA48 + HA35; *crtZ2*: HA34 + HA45; *crtW2*: HA46 + FpW4; *crtZ-L*: HA34 + HA49; *L-crtW*: HA50 + FpW4; *idi*: HA67 + HA68; *crtBI*: HA69 + HA70; *idi-idsA*: HA67 + NH56; *idsA-L*: NH55 + NH57; *L-crtBI*: HA71 + HA70; *idi-idsA-L*: HA67 + NH57. All genes were cloned with their native ORF sequences from gene donors. For transcriptional fusions of crtZ/crtW genes the artificial linker sequence AACTGCCACACGAAC was used and the consensus ribosome binding sequence with optimal spacing (GAAAGGAGGCCCTTCAG) was used for each gene. For pECXT99A derivatives, 15 bp artificial linker sequences were used and the consensus ribosome binding sequence with optimal spacing was inserted in front of *idi*, *idsA*, and *crtBI*. All relevant oligonucleotides used for cloning are included in Table 2. PCR products were cloned into the digested vectors by Gibson method [45]. Colony-PCR was performed with standard vector oligonucleotides (pSH1: PD5 + 582; pECXT99A: pEC-fw + 582).

### 4.4. Carotenoid Quantification

Carotenoid production was analyzed by HPLC analysis. First, carotenoids were extracted from the cell fraction of the cultivation broth using a methanol:acetone (7:3) mixture. Extraction was performed at 60 °C and 600 rpm for 15 min. After centrifugation at 14,000 rpm and 10 min the supernatant was used for high performance liquid chromatography (HPLC). The Agilent 1200 series system (Agilent Technologies, Waldbronn, Germany) was used with a reversed phase column system. Carotenoids were detected with a diode array detector (DAD) through recording of the UV/visible (Vis) spectrum. For quantification the extracted wavelength chromatogram at λ_max_ 470 nm was used. Calibration curves were generated with standard substances: β-carotene, canthaxanthin and astaxanthin (Sigma-Aldrich). The column system consisted of a precolumn (LiChrospher 100 RP18 EC-5, 40 × 4 mm) and a main column (LiChrospher 100 RP18 EC-5, 125 × 4 mm). Methanol (A) and methanol:water (9:1) (B) were used as the mobile phase. A gradient at a flow rate of 1.5 mL/min was used as the following; 0 min B: 0%, 10 min B: 100%, 32.5 min B: 100%.

## Figures and Tables

**Table 1:** Strains and plasmids used in this study.

Strain	Characteristics	Reference
***C. glutamicum* strains**		
WT	Wild type, ATCC 13032	[40]
MB001	prophage cured, genome reduced ATCC 13032	[39]
BETA4	MB001 derivative with deletion of *crtYEb* (cg0717-0719) and *crtR* (cg0725) and integration of P*tuf-dxs*, P*tuf-crtEBI*, P*tuf*-*crtY_Pa_*	[13]
ASTA1	BETA4 derivative with pSH1-*crtW_Fp_* and pECXT99A-*crtZ_Fp_*	[13]
ASTA*	BETA4 derivative with pSH1-*crtZ~W_Fp_*	This work
**Other strains**		
*E. coli* DH5α	F-*thi-*1 *endA1 hsdr17*(r-, m-) *supE44* Δ*lacU169* (Φ80*lacZ*ΔM15) *recA1 gyrA96*	[41]
*Pantoea ananatis*	Wild type, ATCC 33244, DSM 17873, Z96081	[42]
*Fulvimarina pelagi*	Wild type, ATCC BAA-666, DSM 15513, AY178860	[43]
**Plasmids**		
pECXT99A (pECXT)	Tet^R^, *P_trc_lacI^q^*, pGA1 *oriV_Cg_*, *C. glutamicum*/*E. coli* expression shuttle vector	[44]
pECXT-*idi*	pECXT99A derivative for IPTG-inducible expression of *idi* (cg2531)	This work
pECXT-*idsA*	pECXT99A derivative for IPTG-inducible expression of *idsA* (cg2384)	This work
pECXT- *crtBI*	pECXT99A derivative for IPTG-inducible expression of *crtBI* (cg0721/0720)	This work
pECXT-*idi-idsA*	pECXT99A derivative for IPTG-inducible expression of *idi* (cg2531) and *idsA* (cg2384)	This work
pECXT-*idsA-crtBI*	pECXT99A derivative for IPTG-inducible expression of *idsA* (cg2384) and *crtBI* (cg0721/0720)	This work
pECXT-*idi-idsA-crtBI*	pECXT99A derivative for IPTG-inducible expression of *idi* (cg2531), *idsA* (cg2384) and *crtBI* (cg0721/0720)	This work
pSH1	Km^R^, *P_tuf_*, pHM519 *oriV_Cg_*, *C. glutamicum*/*E. coli* expression shuttle vector	[13]
pSH1-*crtW-crtZ*	pSH1 derivative for constitutive expression of the artificial operon comprising *crtW* and *crtZ* from *F. pelagi*	This work
pSH1-*crtW~Z*	pSH1 derivative for constitutive expression of *crtW~Z* encoding for a fusion protein comprising CrtW and CrtZ from *F. pelagi*	This work
pSH1-*crtZ-crtW*	pSH1 derivative for constitutive expression of the artificial operon comprising *crtZ* and *crtW* from *F. pelagi*	This work
pSH1-*crtZ~W*	pSH1 derivative for constitutive expression of *crtZ~W* encoding for a fusion protein comprising CrtZ and CrtW from *F. pelagi*	This work

**Table 2:** Oligonucleotides used in this study.

Oligo-nucleotide	Target	Sequence (5’ → 3’)
FpW1	Wfw1	CATGCCTGCAGGTCGACTCTAGAGGAAAGGAGGCCCTTCAGATGACCCTCAGCCCAACCTC
HN05	Wrv1	GTTCGTGTGGCAGTTTTAGGACTGGCGAGTATGCG
HN06	Zfw1	AACTGCCACACGAACGAAAGGAGGCCCTTCAGATGACGATCTGGACTCTCTACTAC
HA35	Zrv1	ATTCGAGCTCGGTACCCGGGGATCTTACCGAACCGGCGCGT
HA47	Wrv-L	CGGAACCGCCACCGCCGGACTGGCGAGTATG
HA48	L-Zfw	GGCGGTGGCGGTTCCGGCGGTCCAGGTTCCACGATCTGGACTCTCTACTAC
HA34	Zfw2	CATGCCTGCAGGTCGACTCTAGAGGAAAGGAGGCCCTTCAGATGACGATCTGGACTCTCTACTAC
HA45	Zrv2	GTTCGTGTGGCAGTTTTACCGAACCGGCGCGT
HA46	Wfw2	AACTGCCACACGAACGAAAGGAGGCCCTTCAGATGACCCTCAGCCCAACCTC
FpW4	Wrv2	ATTCGAGCTCGGTACCCGGGGATCTTAGGACTGGCGAGTATGCG
HA49	Zrv-L	CGGAACCGCCACCGCCCCGAACCGGCGCGT
HA50	L-Wfw	GGCGGTGGCGGTTCCGGCGGTCCAGGTTCCACCCTCAGCCCAACCTC
HA67	Idifw	ATGGAATTCGAGCTCGGTACCCGGGGAAAGGAGGCCCTTCAGATGTCTAAGCTTAGGGGCATGAC
HA68	Idirv	GCATGCCTGCAGGTCGACTCTAGAGGATCTTACTCTGCGTCAAACGCTTC
HA69	CrtBIfw	ATGGAATTCGAGCTCGGTACCCGGGGAAAGGAGGCCCTTCAGATGACACACCAAAATTCGCC
HA70	CrtBIrv	GCATGCCTGCAGGTCGACTCTAGAGGATCTTAATGATCGTATGAGGTCTTTTGAG
NH56	idsArv	GCATGCCTGCAGGTCGACTCTAGAGGATCTTACATCCGACGTTCGGTTGA
NH55	idsAfw	ATGGAATTCGAGCTCGGTACCCGGGGAAAGGAGGCCCTTCAGATGAGCAGTTTCGATGCCCA
NH57	idsA-rv-L	GTTCGTGTGGCAGTTTTACATCCGACGTTCGGTTGA
HA71	L-CrtBIfw	AACTGCCACACGAACGAAAGGAGGCCCTTCAGATGACACACCAAAATTCGCC
PD5		ACCGGCTCCAGATTTATCAG
582		ATCTTCTCTCATCCTCCA
pEC-fw		AATACGCAAACCGCCTCTCC
